# Self-efficacy, procrastination, and burnout in post-secondary faculty: An international longitudinal analysis

**DOI:** 10.1371/journal.pone.0226716

**Published:** 2019-12-30

**Authors:** Nathan C. Hall, So Yeon Lee, Sonia Rahimi

**Affiliations:** Department of Educational and Counselling Psychology, McGill University, Montreal, Canada; Universitat de Valencia, SPAIN

## Abstract

To address the present research gap on relations between motivational beliefs, self-regulation failure, and psychological health in post-secondary faculty, the present study used associative latent growth modeling to longitudinally examine relationships between self-efficacy, procrastination, and burnout (emotional exhaustion) in faculty internationally. Findings from 3,071 faculty participants (70% female, 69 countries) over three time points (5–6 month lags) showed greater self-efficacy at baseline to correspond with lower procrastination and burnout, and procrastination to be positively related to burnout (intercepts). Growth analyses additionally revealed stronger relations between increases in self-efficacy, procrastination, and burnout over time (slopes). Supplemental cross-lagged analyses provided causal evidence of burnout as an antecedent of self-efficacy and procrastination, underscoring intervention and policy efforts to address overwork and exhaustion in post-secondary faculty.

## Introduction

Over the past 20 years, faculty at post-secondary institutions internationally have experienced rising levels of stress and burnout due to increasing demands for quality instruction, research excellence, and service contributions without commensurate increases in institutional support [1–3]. Beyond the notable impact of institutional demands (e.g., teaching [4]; research [5]) and support (e.g., teaching [6–7]), existing faculty development research has also explored the role of psychological factors in well-being levels (for reviews, see [8–9]). With respect to motivational variables, perceptions of competence in post-secondary faculty has received particular attention given findings showing higher levels of perceived competence (e.g., self-efficacy, control) to consistently correspond with greater teaching success [10–11], research productivity [12–13], and well-being [5,14]. Although adaptive self-regulation strategies have received less empirical attention in faculty research to date (e.g., humor coping [15]), self-regulation failure has been repeatedly examined in relation to both faculty productivity (e.g., writing procrastination [16–17]) and burnout (e.g., inability to cope [1]). However, there to date exists no published research exploring longitudinal relations between faculty motivation, self-regulation, and well-being thereby limiting our understanding of potential causal relationships between these critical psychological variables. To address this research gap, the present longitudinal study evaluated faculty perceptions of self-efficacy, procrastination, and burnout (emotional exhaustion) at three points using latent growth and cross-lagged structural equation models to provide an in-depth analysis of hypothesized relations between these variables with respect to valence, magnitude, as well as causality.

### Motivation in faculty: Self-efficacy beliefs

Motivational variables have consistently been found to correspond with productivity and job satisfaction in post-secondary faculty (e.g., perceived value [18]; perceived competence [19]), with perceptions of self-efficacy having been previously examined in relation to both faculty employment and well-being outcomes. The theoretical construct of self-efficacy is derived from social-cognitive theory [20–21] and is defined as beliefs or confidence regarding one’s capability to manage and perform specific behaviors. Self-efficacy has long been found to be a strong predictor of performance outcomes across academic populations over and above the effects of prior achievement, including K-12, undergraduate, and graduate students (e.g., for reviews, see [22–23]) as well as K-12 teachers [24]. With respect to post-secondary faculty, international research over the past three decades has also explored this social-cognitive construct as reflective of faculty beliefs in their ability to teach (e.g., The Netherlands [25]; China [26]), conduct research (e.g., Turkey [27]; U.S. [28]), and engage in service (e.g., public outreach among U.S. faculty [29–30]), among other academic activities (e.g., administration and consulting, Australia [31]; computing, Nigeria [32]; see also [33]).

In addition to recent qualitative work investigating critical social-environmental contributors to faculty self-efficacy beliefs (e.g., mentorship, collegiality; Australia [34]; Mexico [35]; U.S. [10]), research on faculty self-efficacy has consistently explored demographic antecedents including gender, rank, and discipline. Whereas some studies report no gender differences in teaching or research self-efficacy [31,36], other findings persistently show male faculty to report higher levels of self-efficacy for research and service than females [26,28,37–38]. Similar to findings on teaching self-efficacy among K-12 educators [39], existing research has also shown faculty self-efficacy beliefs for research to be higher among senior relative to junior faculty members [31,36]. Prior studies have further examined faculty self-efficacy beliefs specific to a given discipline (e.g., STEM [30]; foreign language instruction [27,35]), with scattered findings showing faculty self-efficacy levels to be more problematic in specific disciplines relative to others (e.g., education [33]; social sciences [26]; accounting [31]).

Longitudinal research has also been conducted to examine changes in faculty self-efficacy levels, specifically to evaluate the cognitive effects of professional development initiatives. Alongside multiple intervention studies showing pedagogical training programs to increase teaching self-efficacy in faculty internationally (U.S. [40]; Finland [41–42]; India, South Africa [43]), recent findings further show faculty development efforts to improve self-efficacy for research self-efficacy (Turkey [27]) and service over time (i.e., for engaging in gender-equity-promoting behaviors; U.S. [44]). Faculty self-efficacy has also been repeatedly assessed in relation to critical outcome measures, with findings showing greater teaching self-efficacy to correspond with indicators of teaching effectiveness (U.K. [45]; China [46]), and higher research self-efficacy to be related to greater research productivity (e.g., Australia [31,37,47]; U.S. [28,48]). However, beyond recent cross-sectional studies exploring relations between faculty self-efficacy and emotional well-being variables (teaching-related emotions [46]; perceived stress [49]), research on how self-efficacy intersects with psychological health in post-secondary faculty internationally is currently lacking.

### Self-regulation in faculty: Procrastination behaviors

In addition to the role of self-efficacy beliefs in faculty development, research has also begun to explore the utility of higher-order self-regulation constructs to account for faculty performance and well-being outcomes. Nevertheless, despite a voluminous literature with students on the academic implications of self-regulation strategies [50], self-regulation among post-secondary educators with respect to teaching and research [51] or corresponding emotions (e.g., emotional labor [52]; humor coping [15]) has rarely been examined. However, notable exceptions to this research gap include scattered studies assessing *self-regulation failure* in post-secondary faculty with respect to global beliefs in one’s inability to cope [1,5], and an emerging body of research on faculty procrastination. More specifically, following from studies that examined how faculty perceive student procrastination [53–54], or how their instructional methods could impact student procrastination [55], limited research has also investigated the characteristics and correlates of academic procrastination behaviors in post-secondary faculty [16–17].

Procrastination is commonly defined as a dysfunctional phenomenon whereby individuals needlessly delay a task or an action despite expected negative consequences [56,57], with this behavior typically characterized as a failure of self-regulation toward a desired goal [57–61]. More specifically, procrastination researchers suggest that this maladaptive behavior can represent self-regulation failure in two ways: underregulation and/or misregulation. Whereas the *underregulation hypothesis* asserts that procrastination can result from poor behavioral or motivational self-regulation (e.g., ineffective work strategies, insufficient self-control or persistence [57,59,62–63]), the *misregulation hypothesis* proposes that individuals may instead prioritize downregulating negative emotions (e.g., anxiety) through procrastination over accomplishing an achievement goal [58,64–66]. Accordingly, findings in academic contexts have consistently found higher levels of procrastination in students to correspond with poorer performance [67–69] and self-efficacy levels [70–71], as well as lower levels of emotional well-being [72–74].

Limited existing research on procrastination in post-secondary faculty similarly suggests that this behavior is not only regularly experienced but may also correspond with maladaptive performance and psychological outcomes. Early findings showed new faculty in the U.S. to report frequently procrastinating on scholarly writing tasks (e.g., research manuscripts) and unintentionally delaying writing activities due to busyness with other academic responsibilities [17]. These descriptive results were followed up by findings showing a structured intervention that addressed binge writing to improve writing productivity in faculty who reported scholarly writing as a challenge [17,75]. Research by Ackerman and Gross [16] further delineated the specific task components that elicit procrastination in academic staff, with an online survey of faculty across the U.S. showing procrastination to be most frequent when tasks were perceived as ambiguous, difficult, or in conflict with competing deadlines. Most recently, findings from Sharma and Kaur [49] with female college and university lecturers in India showed occupational self-efficacy and procrastination behaviors to be strongly interrelated (*r* = -.67), with both variables further demonstrating significant correlations of equivalent magnitude with occupational stress (self-efficacy: *r* = -.58; procrastination: *r* = .62). Thus, despite emerging research highlighting the potential complementary utility of academic procrastination for predicting faculty development outcomes, in addition to more commonly assessed motivational beliefs, empirical research on procrastination in relation to academic competencies and psychological well-being in faculty members is unfortunately scarce.

### The present research

To address the notable research gaps on relations between self-efficacy, procrastination, and well-being in post-secondary faculty, the present longitudinal study examined these critical psychological variables in an international sample using both latent growth and cross-lagged models to provide lacking large-scale empirical evidence as to respective valences, magnitudes, and causality. Expanding on previous studies exploring cross-cultural differences in faculty self-efficacy (e.g., Australia vs. U.K. [33]), participants were recruited from several countries to afford a representative sample of academics internationally. Consistent with prior intervention research on faculty self-efficacy employing longitudinal designs [41–42], this study further administered reliable measures of self-efficacy, procrastination, and burnout (emotional exhaustion) across three study phases to elucidate potential causal relationships. Moreover, following from recent research efforts to examine the effects of faculty self-efficacy and procrastination on not only performance but psychological health (i.e., emotions [46]; stress [49]), the present research also specifically assessed an internationally validated measure of emotional exhaustion to optimally investigate how motivational and self-regulation factors intersect with a critical psychological indicator of occupational burnout.

#### Hypothesis 1: Self-efficacy and procrastination

Higher levels of self-efficacy intercepts and slopes were hypothesized to correspond with lower procrastination intercepts and slopes, respectively. This hypothesis was derived from assumed positive relations between self-efficacy and self-regulation competencies as outlined in Bandura’s [21] theory (for reviews of the hypothesized role of self-efficacy in procrastination relative to competing motivational constructs, see [57,76]). This hypothesis was additionally informed by the *underregulation* hypothesis which asserts that procrastination can result from lacking academic competencies [57,62] and by empirical studies showing self-efficacy to be negatively correlated with procrastination in students [61,70–71,77–79] and post-secondary lecturers [49]. Given the exploratory nature of the study, differential magnitudes of relations between study variables, patterns of relations between intercepts and slopes of different variables, and directions of causality between study variables were not proposed.

#### Hypothesis 2: Self-efficacy and burnout

Higher levels of self-efficacy at baseline (intercepts) and over time (slopes) were further hypothesized to correspond with lower intercepts and slopes for the burnout subscale of emotional exhaustion, respectively. This hypothesis was informed by the emotional benefits of self-efficacy as outlined in Bandura’s [21] social-cognitive theory, and research demonstrating negative relations between self-efficacy and negative affectivity in students [22], K-12 teachers (e.g., burnout [80–82]), and post-secondary faculty (teaching emotions [46]; occupational stress [49]).

#### Hypothesis 3: Procrastination and burnout

Finally, higher levels of procrastination intercepts and slopes were hypothesized to correspond with higher intercepts and slopes for emotional exhaustion, respectively. This hypothesis follows from the *misregulation* hypothesis in which procrastination is proposed as a self-protective response to negative emotions (e.g., anxiety [65–66]), as well as existing findings showing procrastination to correlate positively with negative affectivity in both students [64,83–87] and post-secondary lecturers [49].

## Materials and methods

### Participants and procedure

Faculty participants (*N* = 3,071) employed at post-secondary institutions across 69 countries were recruited predominantly via social media (Facebook: 57.9%, Twitter: 40.2%; blogs/web/email: 1.9%) as part of a three-phase data collection effort examining self-regulation and academic success in higher education [88–90]. Participants’ mean age was 39 years (*SD* = 8.51) and 69.7% of the sample identified as female, with most participants being employed at institutions in the United States (63.7%), United Kingdom (9.4%), Canada (8.2%), Australia (4.4%), and European countries (8.7%). Faculty were employed across ranks (e.g., U.S: 28% contingent, 39% assistant, 22% associate, 11% full; Canada: 25% contingent, 29% assistant, 34% associate, 12% full; U.K: 52% lecturer, 26% senior lecturer, 13% reader, 10% professor; Australia: 11% tutor, 45% lecturer, 28% senior lecturer, 9% reader, 7% professor) and across disciplines (48 in total, including professional, social/natural sciences, humanities), and had been employed as an academic for an average of 7 years (*SD* = 6.55). Participants completed an omnibus online questionnaire at three time points (5–6 month lags) including demographic items (e.g., age, gender, years of employment) followed by self-report measures of procrastination, self-efficacy, and burnout, and were compensated by cash prize draw after each study phase ($500 per phase). Study protocols were approved by the Research Ethics Board at McGill University (REB File #: 261–0115). Sample sizes for each study phase/measure and descriptive statistics for all study variables (averaged scores across scale items) are presented in Table 1.

**Table 1:** Psychometric properties of study variables.

Variable	*n*	*M*	*SD*	α	Items	Range
Time 1						
Self-efficacy	2,553	37.63	6.08	.81	10	1–5
Procrastination	2,308	34.52	11.22	.93	12	1–5
Burnout	2,251	28.91	10.27	.92	7	1–7
Time 2						
Self-efficacy	1,742	38.10	6.31	.84	10	1–5
Procrastination	1,612	34.28	11.07	.93	12	1–5
Burnout	1,576	29.06	10.21	.93	7	1–7
Time 3						
Self-efficacy	1,096	37.83	6.13	.83	10	1–5
Procrastination	1,048	34.07	11.11	.93	12	1–5
Burnout	1,033	28.57	9.92	.93	7	1–7

Data were collected at three time points administered at 5–6 month lags.

### Study measures

#### Self-efficacy beliefs

Informed by Bandura's [21] social cognitive theory, a 10-item, five-point Likert scale developed for this study assessed faculty members' perceived capabilities with respect to prototypic academic responsibilities (α_t1/t2/t3_ = .81/.84/.83; 1 = *strongly disagree* to 5 = *strongly agree*). Consistent with prior assessments of faculty self-efficacy with respect to primary academic tasks (i.e., teaching and research [31,46]), scale items assessed participants’ perceived confidence in their abilities with respect to student instruction and supervision (e.g., “teach effectively”; “have supervised students graduate in a timely manner”) as well as research activities (e.g., “write a literature review paper”; “deliver a public academic presentation”). A composite self-efficacy measure consisting of items pertaining to both teaching and research was assessed in our main analyses due to exploratory factor analyses showing all items to load significantly on a single factor (no rotation; factor loadings ≥ .30; eigenvalue = 3.75) and the domain-general nature of the other main variables (e.g., academic procrastination vs. research or teaching procrastination).

#### Procrastination frequency

To evaluate perceived frequency of procrastination behaviors in faculty participants, a 12-item, five-point Likert measure compiled by Steel (Pure Procrastination Scale, PPS [91]) was administered (α_t1-t3_ = .93; 1 = *strongly disagree* to 5 = *strongly agree*). The PPS represents a composite of top-loading items from multiple domain-general measures of procrastination in adult populations (i.e., Adult Inventory of Procrastination, AIP [92]; Decisional Procrastination Questionnaire, DPQ [93]; General Procrastination Scale, GPS [94]). The PPS has previously been found to demonstrate higher reliability than its constituent measures (e.g., α = .92 [91]) and shows significant relations with established well-being scales (e.g., Satisfaction with Life Scale [95]; *r* = -.41). The scale preamble was modified from the original to encourage faculty respondents to refer specifically to their academic procrastination behaviors (i.e., “The items below concern your everyday academic work experiences"). Sample items include: “I am not very good at meeting deadlines” (AIP); “Even after I make a decision I delay acting upon it” (DPQ); and “In preparation for some deadlines, I often waste time by doing other things” (GPS).

#### Burnout: Emotional exhaustion

To evaluate an indicator of occupational burnout in faculty participants, the seven-item, six-point emotional exhaustion subscale of the Maslach Burnout Inventory (MBI [96]) was administered (α_t1/t2/t3_ = .92/.93/.93; 1 = *never* to 6 = *every day*). The emotional exhaustion MBI subscale was selected based on recent research with K-12 teachers [82] and post-secondary faculty [97] showing this subscale to demonstrate substantially better internal reliability as compared to the depersonalization and personal accomplishment subscales, with the accomplishment subscale also not assessed to prevent redundancy with the self-efficacy measure. The scale preamble was modified to instruct participants to refer specifically to their experiences as a faculty member, with two scale items removed from the original nine-item exhaustion measure following from international MBI validation data showing consistently poor factor loadings for two service profession items pertaining to stress/strain from “working with people” [98]. Sample subscale items include: “I feel emotionally drained from my work”; “I feel fatigued when I get up in the morning and have to face another day at work”; and “I feel I’m working too hard at my job.”

## Results

### Preliminary analyses

#### Participant attrition

Given that substantial attrition is typically observed in longitudinal studies with repeated assessments (e.g., ranging from 30% to 70% [99–100]), preliminary analyses were additionally conducted to assess potential differences in participant demographics and the main study variables (self-efficacy, procrastination, exhaustion at Time 1) as a function of three attrition levels (completed Time 1, Times 1–2, Times 1–3). As outlined in Table 1 (see self-efficacy), 30% attrition was observed from Time 1 to 2, with 37% attrition found from Time 2 to 3 (57% total study attrition). ANOVA results showed faculty who completed only the first phase to report higher emotional exhaustion at Time 1 (*M* = 29.65, *SD* = 10.78) than those who completed two study phases (*M* = 28.23, *SD* = 9.96; Games-Howell contrast), however this effect was notably weak in magnitude *F*(2, 2205) = 3.36, *p* = .035, η_p_^2^ = .003. In contrast, ANOVA and chi-squared analyses showed no statistically significant differences in participants’ gender, age, country (of institution), years of employment, self-efficacy, and procrastination as a function of study attrition, with attrition results overall thus underscoring the representativeness of the total study sample with respect to both key background characteristics and the main study variables.

#### Initial differences

To evaluate the extent to which specific demographic background variables examined in prior faculty research were associated with mean levels or could moderate changes over time in self-efficacy, procrastination, and burnout in the present study, repeated measures ANOVAs were conducted on each variable with gender and country (of institution) as between-subjects variables alongside the within-subjects effect of time. Gender was selected based on prior research consistently showing male faculty to report higher research and service self-efficacy relative to females [26–38]. Country of institution was also examined (i.e., five main countries: U.S., U.K., Canada, Australia, Europe) in that although prior research has investigated faculty self-efficacy and procrastination in various countries (e.g., India, China, U.S., Turkey, Nigeria, Mexico, Australia, U.K., The Netherlands), no research to date has obtained international data affording direct comparisons between more than two countries on these variables (cf. Australia vs. U.K. [33]).

Significant within-subjects effects of time were observed only on self-efficacy (e.g., gender ANOVA: *F*(2,1836) = 6.51, *p* = .002, η_p_^2^ = .006), showing self-efficacy levels to increase slightly from Time 1 to 2 (see Table 1). No within-subjects interactions between time and gender or country were observed and no between-groups country effects were significant. Consistent with prior research on faculty burnout (e.g., emotional exhaustion [8]), significant between-subjects effects showed female faculty to report slightly greater exhaustion levels relative to males, *F*(1,887) = 5.73, *p* = .017, η_p_^2^ = .006. Moreover, whereas prior research on faculty procrastination has not examined gender differences [16–17] or was gender-specific (i.e., females [49]), the present findings also showed female faculty to report consistently lower levels of procrastination relative to males, *F*(1,918) = 7.79, *p* = .005, η_p_^2^ = .008. Nevertheless, due to a lack of significant differences between countries, no moderation effects of gender or country on changes over time, and the gender effects being notably weak in magnitude, these variables were not evaluated as potential confounds or moderators in our main structural equation models.

#### Zero-order correlations

Correlational analyses on averaged scale items for each self-report measure (Table 2) showed moderate to strong intercorrelations among the three assessments of self-efficacy, procrastination, and burnout (.67 < *r*s < .84), particularly between adjacent assessments. Weak correlations in the expected directions were otherwise observed between self-efficacy and procrastination (-.21 < *r*s < -.16) and between procrastination and the emotional exhaustion burnout subscale (.20 < *r*s < .24), with the weakest correlations found between self-efficacy and exhaustion (-.13 < *r*s < -.09). These findings underscore the orthogonality of the faculty motivation, self-regulation, and well-being variables administered in this study and show multicollinearity to not represent a substantial confound in our main structural equation models.

**Table 2:** Zero-order correlations among study variables.

Variable	1	2	3	4	5	6	7	8
1. SE/T1	-							
2. SE/T2	.67	–						
3. SE/T3	.67	.77	–					
4. PR/T1	-.21	-.13	-.16	–				
5. PR/T2	-.16	-.17	-.16	.81	–			
6. PR/T3	-.19	-.18	-.20	.81	.84	–		
7. BU/T1	-.12	-.09	-.08	.23	.23	.23	–	
8. BU/T2	-.10	-.12	-.11	.20	.24	.20	.72	–
9. BU/T3	-.09	-.13	-.11	.21	.20	.24	.70	.76

SE = self-efficacy; PR = procrastination; BU = burnout; T = time of assessment. All zero-order correlations are statistically significant at *p* < .001.

### Main analyses

#### Associative latent growth curve model

**Fig 1:**
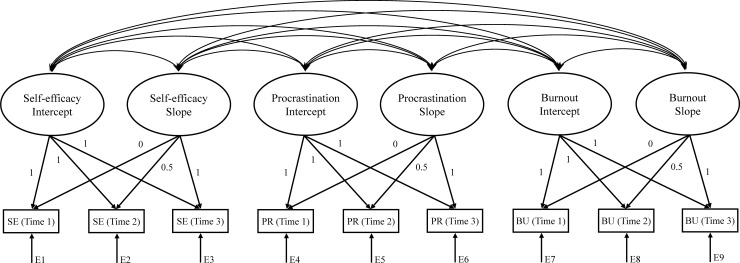
Associative latent growth curve model.

Latent growth curve modeling (LGCM) was conducted via Amos 21.0 software to examine the covariance among intercepts (baselines) and slopes (increases over time) over three assessments of faculty self-efficacy, procrastination, and emotional exhaustion (for more on LGCM protocols, see [101–102]). Missing data were automatically computed using maximum likelihood (ML) estimation [103], with the Comparative Fit Index (CFI), Tucker-Lewis index (TLI), and Root Mean Square Error of Approximation (RMSEA) evaluated as indicators of model fit. The LGCM evaluating hypothesized covariances between self-efficacy, procrastination, and burnout (emotional exhaustion) intercepts and slopes is outlined in Fig 1.

All model fit indices were satisfactory indicating sufficient power to detect differences between the observed data and predicted model [104–105]; CFI = .99, TLI = .98, RMSEA = .03. Inspection of the latent overall mean (*M* = 37.59, *SE* = .12, *p* < .001) and slope mean for self-efficacy (*M* = .38, *SE* = .14, *p* < .01), as well as intercept (σ2 = 27.13, *SE* = 1.43, *p* < .001) and slope variances (σ2 = 10.67, *SE* = 2.64, *p* < .001), revealed both significant individual differences in baseline levels and intra-individual differences in growth in self-efficacy over time. For procrastination (latent overall *M* = 34.53, *SE* = .23, *p* < .001), significant intercept variance further revealed individual differences between faculty on this variable (σ2 = 101.83, *SE* = 4.35, *p* < .001). For the burnout measure of emotional exhaustion (latent overall *M* = 29.02, *SE* = .21, *p* < .001), the variances for both the intercept (σ2 = 81.40, *SE* = 4.09, *p* < .001) and slope (σ2 = 25.64, *SE* = 7.11, *p* < .001) further suggested both initial differences as well as intra-individual differences in growth in exhaustion over time. Slope means for procrastination (*M* = -.10, *SE* = .20, *p* = .635) and burnout (*M* = -.09, *SE* = .34, *p* = .679) were not significant.

Covariances between the intercepts and slopes for each of the study variables are outlined in Table 3. With respect to relations between intercepts and slopes for the same variable, although these covariances were not significant for self-efficacy and procrastination, it was significant for emotional exhaustion (-.20, *p* = .034) in showing faculty with initially high levels of exhaustion to demonstrate a lower rate of increase in exhaustion over the subsequent two assessments. Concerning covariances between *intercepts* for the study variables (|.18|-|.28|), baseline levels of self-efficacy and procrastination, self-efficacy and exhaustion, as well as procrastination and exhaustion were found to significantly covary in the expected directions. However, even stronger relations in the hypothesized directions were observed between the *slopes* for each of the study variables (|.21|-|.46|), underscoring the correspondence between faculty self-efficacy, procrastination, and burnout not only at baseline but also in terms of shared trajectories over time. Weak yet significant covariances between the slope for self-efficacy and the intercepts for procrastination and exhaustion were also observed, suggesting that faculty with poorer procrastination and emotional exhaustion levels at baseline were more likely to show increases in self-efficacy over time, suggesting potential floor effects or regression to the mean.

**Table 3:** Latent covariances between intercepts and slopes of study variables.

	Self-efficacy		Procrastination		Burnout	
	Intercept	Slope	Intercept	Slope	Intercept	Slope
Self-efficacy						
Intercept	–					
Slope	-.06	–				
Procrastination						
Intercept	-.26***	.12**	–			
Slope	.11	-.46***	-.02	–		
Burnout						
Intercept	-.18***	.13**	.28***	.00	–	
Slope	.03	-.21***	-.06	.41***	-.20*	–

*p < .05.

**p ≤ .01.

***p < .001

#### Supplemental cross-lagged analyses

As a follow-up to the associative LGCM findings showing substantial relations between the study variables over time, exploratory cross-lagged structural equation models were further conducted to examine potential causal relations between self-efficacy, procrastination, and burnout (emotional exhaustion) in our faculty sample. Three cross-lagged analyses were performed, each contrasting two study variables over three time points (self-efficacy vs. procrastination, self-efficacy vs. burnout, procrastination vs. burnout). Autoregressive paths between each assessment for the same variable were modelled (e.g., self-efficacy at Time 1 to Time 2), with covariances additionally modelled between variables within the same assessment period (e.g., self-efficacy at Time 1 with procrastination at Time 1) and between error terms for equivalent item parcels (e.g., self-efficacy Time 1 parcel 1 with self-efficacy Time 2 parcel 1). Due to these supplemental analyses affording greater specificity than the LGCM with respect to manifest indicators (by concurrently examining two vs. three study variables), scale items were parceled in a sequential manner (items summed with subsequent items for unidimensional variables; see [106–107]) to evaluate the latent self-efficacy, procrastination, and exhaustion variables with roughly equivalent numbers of manifest indicators (5, 4, and 3 parcels respectively). Given the highly conservative nature of cross-lagged analyses in controlling for autoregressive paths, statistically significant cross-lagged parameters (e.g., self-efficacy at Time 1 to procrastination at Time 2) were not expected to be large in magnitude but instead suggestive of the potential existence and valence of causal relationships [108].

**Fig 2:**
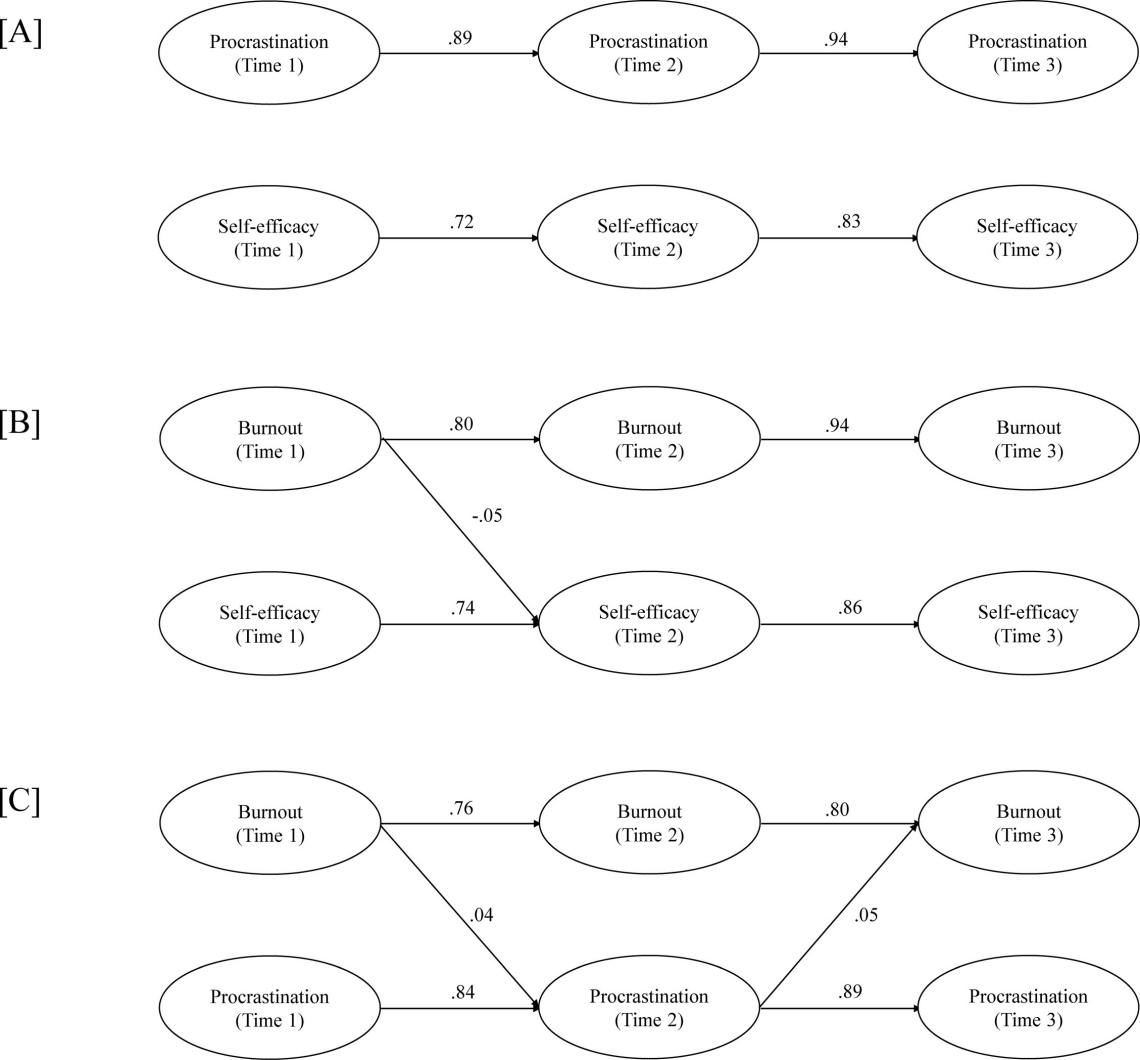
**Cross-lagged Results for [A] Self-efficacy and Procrastination, [B] Self-efficacy and Burnout, [C] Procrastination and Burnout.** Only significant standardized estimates for directional auto-regressive and cross-lagged paths are displayed.

The first analysis evaluating potential directional relations between self-efficacy and procrastination (see Fig 2, Panel A) showed adequate model fit (CFI = .90, TLI = .86, RMSEA = .06) and no significant cross-lagged effects between the three time points. The second analysis evaluating self-efficacy and burnout (emotional exhaustion) relations (Fig 2, Panel B) demonstrated mediocre model fit (CFI = .82, TLI = .75, RMSEA = .09), and revealed a weak negative effect of exhaustion at Time 1 on self-efficacy at Time 2 (*β* = -.05, *p* = .011). Finally, the analysis of procrastination and exhaustion relations over time showed satisfactory model fit (CFI = .98, TLI = .98, RMSEA = .03), with two significant directional paths between the study variables being observed (Fig 2, Panel C). Although the cross-path parameters were weak in magnitude, a significant bidirectional pattern of relations showed greater burnout at Time 1 to lead to higher levels of procrastination at Time 2 (*β* = -.04, *p* = .038) that, in turn, predicted greater emotional exhaustion at Time 3 (*β* = -.05, *p* = .043). The supplemental cross-lagged findings thus suggest that beyond the notable stability of the self-report measures of faculty self-efficacy, procrastination, and burnout over time (autoregressive paths within variables), specific directions and valences of causal relations may also be inferred (weak yet significant cross-lagged paths between variables). More specifically, higher levels of emotional exhaustion in faculty at Time 1 were found to predict poorer levels of both self-efficacy and procrastination at Time 2, with lower procrastination levels at Time 2 further contributing to exhaustion at Time 3.

## Discussion

The present study findings contribute to existing research on motivational beliefs, self-regulation failure, and psychological health in faculty by examining three quintessential constructs reflecting each psychological domain, namely self-efficacy, procrastination, and burnout (emotional exhaustion). By way of large-scale international recruitment and longitudinal assessments, the present data afforded not only an in-depth analysis of baseline covariance but also shared trajectories in changes over time in these key psychological variables. Concerning relations between self-efficacy and procrastination, our LGCM results provide empirical support for *Hypothesis 1* in showing higher faculty self-efficacy to correspond with lower procrastination not only at baseline (intercepts) but especially over time (slopes), with increases in self-efficacy found to correspond with a lower likelihood of increased procrastination over a one-year period. These results thus underscore the utility of Bandura’s [21] social learning theory for explaining self-regulation behavior in faculty populations and are consistent with the underregulation hypothesis (procrastination as self-regulation failure [57,62]) as well as recent findings showing negative cross-sectional relations between self-efficacy and procrastination among female faculty [49].

The present LGCM findings additionally provide support for *Hypothesis 2* in showing greater faculty self-efficacy to further correspond with lower levels of emotional exhaustion at baseline as well as a significantly lower likelihood of increased exhaustion over time. Accordingly, these findings again support the use of Bandura’s [21] social-cognitive theory to account for psychological well-being in post-secondary faculty, and are consistent with recent cross-sectional findings with academic staff showing greater self-efficacy to correlate with more positive emotions [46] and lower occupational stress [49]. Our latent growth results also supported *Hypothesis 3* such that greater procrastination at baseline, and especially over time, was found to consistently correspond with higher exhaustion levels in our faculty sample. These findings are thus additionally aligned with the misregulation hypothesis (e.g., procrastination as emotion regulation [65–66]) as well as recent cross-sectional findings with female faculty [49].

### Study limitations and implications

Despite consistent empirical support for each of the study hypotheses, it is important to understand the present findings in the context of limitations that could impact replicability and generalizability. First, as has long been suggested in research on the domain-specificity of motivation measures [109], observed relations in this study may be underestimated due to our study measures being global in nature as opposed to differentiating between academic tasks (e.g., faculty self-efficacy for research vs. teaching [31,46]). Second, whereas the present measure of faculty self-efficacy assessed both teaching and research competencies, it did not address service responsibilities (e.g., public scholarship [29]) thus warranting replication with respect to this third critical set of academic tasks. Third, despite preliminary analyses suggesting a lack of systematic attrition as a function of demographic and psychological factors, it is nevertheless possible for missing data to bias latent growth results [110] with faculty who persisted with study completion (or the academic profession) over the one-year study period possibly demonstrating academic “survival bias” [111].

A fourth limitation of this study concerns the exploratory cross-lagged analyses in that although they provide preliminary evidence of directional relationships between the faculty self-efficacy, procrastination, and exhaustion, the magnitudes of these relations were notably weak requiring further research to obtain and triangulate stronger empirical evidence of causal links (e.g., qualitative interviews, observational data, experimental methods). Finally, it also possible that due to the use of social media as a participant recruitment method (i.e., Facebook, Twitter), the present sample may not be representative of faculty who do not regularly use social media (e.g., an estimated 56% of faculty do not use Twitter [112]) with greater attrition rates observed in studies with online recruitment having also potentially contributed to Type I error (e.g., MTurk [113]). However, these recruitment concerns are mitigated in that academics tend to engage regularly with the primary social media platform used for recruitment (e.g., over 50% of academics in the social sciences, arts, and humanities report using Facebook on a regular basis [114]) with our preliminary analyses also showing no differences in gender, age, country, years of employment, self-efficacy, or procrastination due to attrition. Moreover, emerging research further demonstrates largely equivalent (or even superior) validity of responses obtained from participants recruited via social media vs. in-person methods [115].

These limitations notwithstanding, these findings also contribute to existing theories relevant to faculty motivation, self-regulation, and burnout in the following ways. For example, to the extent that greater emotional exhaustion is indicative of excessive work requirements, these results suggest that the effects of context (exhaustion) on motivation (self-efficacy) may be stronger than vice versa, thus clarifying the proposed bidirectional relationship between these variables in Bandura’s [20] social learning theory (i.e., triadic reciprocity) when applied to faculty. Similarly, our cross-lagged findings provide stronger support for the misregulation hypothesis than the underregulation hypothesis [65] suggesting that procrastination may represent a self-protective response to threats to emotional well-being in faculty. Similarly, to the extent that self-efficacy represents an internal psychological resource, and procrastination may imply cognitive disengagement, these findings suggest that the well-known Job Demands-Resources model of occupational burnout [116] may be optimally adapted to faculty populations by examining disengagement as an outcome as opposed to a correlate of burnout experiences.

Concerning practical implications of the study findings, beyond our LGCM analyses demonstrating significant relations over time between our study variables, our cross-lagged analyses further suggest burnout to be more likely to predict subsequent self-efficacy and procrastination than vice versa. Although this effect was weak in magnitude and exploratory in nature, it may nevertheless help to inform orientation and intervention efforts for faculty moving forward if replicated in future studies. More specifically, whereas existing professional development efforts to date aimed at improving faculty self-efficacy beliefs [27,41–42] or reducing procrastination [17,75] show promise with respect to performance and productivity gains, increased institutional efforts to reduce known antecedents of emotional exhaustion due to overwork (e.g., teaching demands [97]; research pressures [5]; for a review, see [8]) may be more effective for improving psychological health in faculty internationally.
